# Multimodality Imaging in Hypertrophic Cardiomyopathy: From Phenotyping to Precision Clinical Management

**DOI:** 10.3390/diagnostics16193149

**Published:** 2026-09-28

**Authors:** Niya Mileva, Panayot Panayotov

**Affiliations:** 1 SHATC Medica Cor, Riga 35, 7013 Ruse, Bulgaria; 2 Medical University of Pleven, 5800 Pleven, Bulgaria; 3 Faculty of Public Health and Health Care, Ruse University “Angel Kanchev”, 7017 Ruse, Bulgaria

**Keywords:** hypertrophic cardiomyopathy, multimodality imaging, echocardiography, cardiac magnetic resonance, cardiac computed tomography, positron emission tomography, myocardial fibrosis, risk stratification, coronary microvascular dysfunction, precision cardiology

## Abstract

Hypertrophic cardiomyopathy (HCM) is a genetically and phenotypically heterogeneous myocardial disease characterized by unexplained left ventricular hypertrophy and a broad spectrum of clinical manifestations, ranging from an asymptomatic phenotype to heart failure, atrial and ventricular arrhythmias, sudden cardiac death, and advanced heart failure. Accurate diagnosis and risk stratification therefore require complex assessment. Multimodality cardiovascular imaging provides complementary structural, functional, and tissue characterization that cannot be obtained using a single imaging technique. Transthoracic echocardiography remains the cornerstone of initial diagnosis, hemodynamic assessment, and longitudinal follow-up, while advanced echocardiographic techniques provide additional information regarding myocardial and left atrial mechanics. Cardiac magnetic resonance (CMR) has become central to phenotypic characterization and tissue assessment. Cardiac computed tomography offers complementary high-resolution anatomical information, particularly for coronary assessment and procedural planning, whereas nuclear imaging, provides insights into coronary microvascular dysfunction, myocardial perfusion, and selected phenocopies such as transthyretin cardiac amyloidosis. Multimodality imaging also facilitates differentiation of HCM from other conditions such as athlete’s heart, hypertensive heart disease, and infiltrative cardiomyopathies. Integration of imaging biomarkers with clinical and genetic information may improve prediction of adverse outcomes. Thus, multimodality imaging should be regarded not as a collection of competing techniques but as an integrated framework for precision diagnosis, risk stratification, therapeutic planning, and longitudinal management of patients with HCM.

## 1. Introduction

Hypertrophic cardiomyopathy (HCM) is the most common inherited cardiomyopathy and is characterized by unexplained left ventricular (LV) hypertrophy in the absence of abnormal loading conditions sufficient to account for the degree of hypertrophy [1]. The estimated prevalence of HCM is approximately 1:500 in the general population, although contemporary studies incorporating genotype-positive individuals and advanced imaging suggest that the true prevalence may be higher. HCM is associated with substantial clinical heterogeneity, ranging from asymptomatic individuals with normal life expectancy to patients presenting with heart failure, atrial fibrillation, ventricular arrhythmias, sudden cardiac death (SCD), or advanced heart failure requiring transplantation [2,3,4].

The genetic basis of HCM is complex and is mostly related to pathogenic variants affecting sarcomeric proteins involved in cardiac contraction and force generation [5]. The disease exhibits marked genetic and phenotypic variability, with incomplete penetrance, age-dependent expression, and variable clinical manifestations even among individuals carrying the same pathogenic variant [6].

The clinical expression of HCM is highly heterogeneous and reflects complex interactions between genetic background, myocardial remodelling, fibrosis, arrhythmogenic substrate formation, and environmental factors. Morphological patterns vary considerably, including asymmetric septal hypertrophy, reverse curvature septal morphology, concentric hypertrophy, mid-ventricular obstruction, and isolated apical hypertrophy. Beyond increased myocardial thickness, disease progression is characterized by diastolic dysfunction, left atrial remodelling, myocardial fibrosis, microvascular dysfunction, and in advanced stages, LV systolic impairment. These diverse phenotypes contribute to the complexity of diagnosis, prognostication, and therapeutic decision-making [2,3].

Imaging plays a central role throughout the clinical course of HCM, from initial diagnosis and phenotypic characterization to risk stratification and treatment planning. Transthoracic echocardiography remains the first-line imaging technique due to its widespread availability, ability to assess cardiac morphology and function, and unique capacity to evaluate dynamic LV outflow tract (LVOT) obstruction at rest and during physiological provocation [7].

Cardiac magnetic resonance (CMR) has emerged as a cornerstone of contemporary HCM evaluation by providing high-resolution assessment of ventricular morphology, accurate quantification of myocardial mass and volumes, and comprehensive tissue characterization [8]. CMR also improves recognition of atypical phenotypes, including apical HCM, LV aneurysm formation, and myocardial involvement in phenocopies [9].

Although echocardiography and CMR represent the foundation of HCM imaging, other modalities provide complementary information in selected clinical scenarios. Cardiac computed tomography (CT) offers excellent spatial resolution for coronary assessment, anatomical characterization, and procedural planning, particularly in patients undergoing septal reduction therapy or those unable to undergo CMR. Nuclear imaging techniques, particularly positron emission tomography (PET), provide unique insights into myocardial perfusion, coronary microvascular dysfunction, and metabolic activity, expanding the diagnostic spectrum beyond structural assessment [10].

The increasing complexity of HCM phenotyping has shifted the diagnostic paradigm from a single-modality approach toward integrated multimodality imaging. Combining the strengths of echocardiography, CMR, CT, and nuclear imaging allows more accurate differentiation between HCM and phenocopies, improves risk assessment beyond traditional clinical variables, and supports individualized therapeutic strategies. Current guidelines emphasize the complementary role of different imaging modalities in diagnosis, family screening, sudden cardiac death risk assessment, and longitudinal disease monitoring [3,11].

The aim of this review is to provide a contemporary overview of the role of multimodality imaging in patients with HCM, focusing on the contribution of echocardiography, CMR, CT, and nuclear imaging to diagnosis, phenotypic characterization, risk stratification, and clinical management. Furthermore, we discuss emerging imaging biomarkers, artificial intelligence applications, and future perspectives toward precision imaging-based care in HCM.

### Literature Search Strategy

Two independent reviewers (N.M. and P.P.) systematically searched PubMed, MEDLINE and SCOPUS. Studies were included in the narrative review according to the following eligibility criteria: (1) studies comprising patients with any form of hypertrophic cardiomyopathy or other conditions that may present as phenocopies, and (2) studies evaluated with non-invasive imaging.

The main search was performed with the following Medical Subject Headings (Mesh) and free-text terms:

(“Cardiomyopathy” OR “Hypertrophic cardiomyopathy” OR “Hypertensive heart”) AND (“Imaging” OR “Multimodality imaging” OR “Echocardiography” OR “Cardiac magnetic resonance” OR “Computed tomography”] OR “Nuclear Imaging” “Positron emission tomography”). We conducted a modified narrative review according to recent recommendations [5]. The search was performed before July 2026, and all relevant data was derived from research articles, reviews, case reports, etc.

## 2. Imaging Targets in Hypertrophic Cardiomyopathy

The clinical complexity of hypertrophic cardiomyopathy reflects the interaction between myocardial hypertrophy, abnormal ventricular mechanics, tissue remodelling, and progressive structural changes. Contemporary imaging aims not only to confirm the diagnosis but also to characterize disease phenotype, identify prognostically relevant features, and guide therapeutic decision-making (Table 1). Although increased left ventricular wall thickness remains the defining morphological feature of HCM, a comprehensive imaging assessment should evaluate multiple disease substrates, including dynamic obstruction, diastolic dysfunction, mitral valve abnormalities, myocardial fibrosis, microvascular dysfunction, atrial remodelling, apical aneurysm formation, and right ventricular involvement (Figure 1). The integration of these parameters provides a more complete assessment of disease severity and clinical risk than any single imaging marker.

### 2.1. Left Ventricular Hypertrophy

The hallmark of HCM is unexplained LV hypertrophy, traditionally defined as a maximal LV wall thickness ≥ 15 mm in adults in the absence of other loading conditions capable of producing a similar degree of hypertrophy. In individuals with a pathogenic sarcomeric variant or affected first-degree relatives, a lower threshold of 13–14 mm may support the diagnosis when accompanied by additional features [2,3]. Echocardiography remains the initial modality for identifying hypertrophy patterns and measuring maximal wall thickness; however, cardiac magnetic resonance provides superior spatial resolution and more accurate assessment of LV mass, hypertrophy distribution, and atypical phenotypes [12].

The morphological expression of HCM is heterogeneous, including asymmetric septal hypertrophy, reverse curvature septal morphology, concentric hypertrophy, mid-ventricular hypertrophy, and isolated apical involvement. CMR is particularly valuable for identifying apical HCM and localized hypertrophy involving the anterolateral wall or papillary muscles, which may be underestimated by echocardiography [13]. Beyond wall thickness, assessment of LV geometry, cavity size, and remodelling pattern provides important information regarding disease progression and prognosis. Advanced imaging techniques, including three-dimensional echocardiography and CMR-based feature tracking, allow more comprehensive evaluation of ventricular structure and mechanics.

### 2.2. Dynamic Left Ventricular Outflow Tract Obstruction

Dynamic LV outflow tract (LVOT) obstruction is a major pathophysiological feature of HCM and results from complex interactions between basal septal hypertrophy, systolic anterior motion (SAM) of the mitral valve, altered ventricular geometry, and abnormal flow dynamics. Resting LVOT obstruction is generally defined as a peak instantaneous gradient ≥30 mmHg, while gradients ≥ 50 mmHg are considered clinically significant when evaluating candidates for septal reduction therapy [2,3].

Transthoracic echocardiography is the primary imaging modality for assessing LVOT obstruction because it enables real-time evaluation of gradients at rest and during physiological provocation, including Valsalva maneuver and exercise. Exercise stress echocardiography is particularly important in symptomatic patients without significant resting obstruction, as latent obstruction may be unmasked during exertion [14]. CMR provides complementary information by accurately characterizing septal morphology, mitral valve anatomy, papillary muscle abnormalities, and the mechanisms contributing to obstruction. Four-dimensional flow CMR is an emerging technique that allows visualization and quantification of abnormal intracardiac flow patterns, although its routine clinical application remains under investigation [15].

### 2.3. Diastolic Dysfunction

Impaired diastolic function is a fundamental component of HCM and results from myocardial hypertrophy, increased ventricular stiffness, impaired relaxation, and interstitial fibrosis. Elevated LV filling pressures contribute to exercise intolerance, dyspnoea, pulmonary hypertension, and left atrial enlargement [3].

Echocardiography remains central for the evaluation of diastolic function using parameters including mitral inflow velocities, tissue Doppler-derived e′ velocity, E/e′ ratio, left atrial volume, and pulmonary venous flow patterns. Global longitudinal strain and myocardial work analysis provide additional insights into myocardial dysfunction that may precede changes in ejection fraction [16]. CMR contributes through assessment of ventricular volumes, myocardial stiffness surrogates, and tissue characterization. Increased extracellular volume fraction (ECV) and native T1 values reflect diffuse myocardial remodelling and have been associated with impaired ventricular relaxation and adverse clinical outcomes [17].

### 2.4. Mitral Valve Abnormalities

Mitral valve abnormalities are common in HCM and contribute significantly to symptoms and LVOT obstruction. These abnormalities include systolic anterior motion of the mitral valve, elongated mitral leaflets, abnormal papillary muscle insertion, accessory papillary muscles, and altered mitral-septal geometry [18]. Echocardiography provides the primary assessment of SAM, mitral regurgitation severity, and dynamic interaction between the mitral valve and LVOT. Three-dimensional echocardiography improves anatomical characterization by providing detailed assessment of leaflet morphology and subvalvular structures. CMR and cardiac computed tomography (CT) provide complementary anatomical information, particularly when planning surgical intervention or septal reduction procedures [19]. Recognition of mitral valve abnormalities is essential because obstruction may result from mechanisms beyond septal hypertrophy alone, influencing the choice of therapeutic strategy.

### 2.5. Myocardial Fibrosis

Myocardial fibrosis represents one of the most important imaging targets in HCM because it reflects disease progression and contributes to electrical instability. Fibrosis creates an arrhythmogenic substrate by disrupting myocardial architecture and promoting conduction heterogeneity. CMR with LGE is the established non-invasive technique for detecting focal replacement fibrosis. LGE in HCM typically demonstrates a patchy mid-wall distribution within hypertrophied segments, although patterns may vary. Extensive LGE has been associated with increased risk of ventricular arrhythmias, sudden cardiac death (SCD), and heart failure progression [9]. Quantitative assessment of LGE burden, particularly values ≥15% of LV mass, has gained importance in contemporary risk assessment strategies [3]. Parametric CMR techniques, including native T1 mapping and extracellular volume quantification, provide assessment of diffuse interstitial fibrosis and may identify myocardial abnormalities before extensive focal fibrosis develops. Elevated native T1 and ECV values have been associated with adverse remodelling and clinical outcomes, although their integration into routine risk algorithms requires further validation [17].

### 2.6. Coronary Microvascular Dysfunction

Coronary microvascular dysfunction is increasingly recognized as an important component of HCM pathophysiology. Increased myocardial mass, abnormal intramyocardial arteriolar structure, and impaired coronary vasodilatory capacity contribute to myocardial ischemia despite normal epicardial coronary arteries [10]. Positron emission tomography (PET) allows quantitative assessment of myocardial blood flow and coronary flow reserve (CFR). Reduced CFR has been associated with myocardial fibrosis, ventricular dysfunction, heart failure progression, and adverse cardiovascular outcomes [20]. Stress perfusion CMR provides an alternative approach for evaluating myocardial ischemia and microvascular dysfunction, although PET remains the reference method for quantitative assessment of myocardial blood flow. The clinical role of microvascular imaging is evolving and may become increasingly relevant for identifying patients at risk of progressive remodelling.

### 2.7. Left Atrial Remodelling

Left atrial (LA) enlargement and dysfunction are common consequences of chronic elevation of LV filling pressures in HCM. LA remodelling reflects the cumulative burden of diastolic dysfunction and is strongly associated with atrial fibrillation (AF), stroke, and heart failure symptoms [21]. Echocardiographic assessment of LA volume index remains a widely used marker of chronic diastolic burden. More recently, LA strain analysis has emerged as a sensitive marker of early atrial dysfunction and may provide incremental prognostic information beyond LA size [22,23]. CMR provides highly reproducible measurements of LA volumes and function and allows assessment of atrial fibrosis using late gadolinium enhancement techniques, although atrial tissue characterization remains primarily a research application [24].

### 2.8. Apical Aneurysm

LV apical aneurysm represents a distinct HCM phenotype characterized by localized apical thinning and dyskinesia, frequently associated with mid-ventricular obstruction. Although relatively uncommon, apical aneurysms are clinically important because they are associated with ventricular arrhythmias, thrombus formation, embolic events, and SCD [25]. CMR is superior to echocardiography for detecting apical aneurysms due to its ability to accurately visualize the LV apex and characterize associated scar tissue using LGE. Identification of apical aneurysm has become an important component of contemporary HCM risk assessment [26].

### 2.9. Right Ventricular Involvement

Although HCM primarily affects the LV, right ventricular (RV) abnormalities may occur and reflect more advanced myocardial involvement. RV hypertrophy, dilation, systolic dysfunction, and abnormal RV-pulmonary artery coupling have been described in subsets of patients and may contribute to exercise intolerance and adverse outcomes [27]. Echocardiography provides initial assessment of RV size and function, including tricuspid annular plane systolic excursion (TAPSE) and RV strain. CMR remains the reference technique for RV volume and function quantification due to its high reproducibility. RV involvement is not a primary diagnostic criterion for HCM but may provide additional information regarding disease severity and prognosis [28].

## 3. Echocardiography

Echocardiography remains the cornerstone of imaging evaluation in patients with hypertrophic cardiomyopathy and represents the first-line modality for diagnosis, phenotypic characterization, hemodynamic assessment, and longitudinal follow-up [2,3]. Its central role is based on widespread availability, lack of ionizing radiation, relatively low cost, and the ability to provide real-time assessment of cardiac structure and function. Importantly, echocardiography uniquely enables dynamic evaluation of LVOT obstruction during physiological maneuvers and exercise, which is essential because obstruction severity may vary considerably under resting conditions [14]. Although CMR has become increasingly important for tissue characterization and refined phenotyping, echocardiography remains indispensable in routine clinical practice. A comprehensive echocardiographic examination in HCM should include assessment of LV hypertrophy pattern, maximal wall thickness, LVOT gradients, systolic anterior motion (SAM) of the mitral valve, mitral regurgitation, LV systolic and diastolic function, left atrial size, and right ventricular function [29]. Recent advances in echocardiographic techniques, including three-dimensional echocardiography, speckle-tracking strain imaging, LA strain analysis, and myocardial work assessment, have expanded the ability of echocardiography to detect subtle abnormalities beyond conventional parameters.

### 3.1. Conventional Transthoracic Echocardiography

Transthoracic echocardiography (TTE) is the initial imaging modality for suspected HCM and remains the primary technique for establishing the morphological diagnosis. The diagnosis is generally based on the presence of unexplained LV hypertrophy, usually defined as maximal LV wall thickness ≥15 mm in adults, or ≥13 mm in individuals with a pathogenic variant or affected first-degree relative [2,3].

TTE provides essential information regarding the distribution and severity of hypertrophy, including asymmetric septal hypertrophy, concentric hypertrophy, reverse curvature morphology, mid-ventricular hypertrophy, and apical involvement. Measurements should be obtained in multiple imaging planes to avoid underestimation or overestimation of wall thickness, particularly in patients with asymmetric or localized forms of disease. Beyond morphology, conventional echocardiography provides critical hemodynamic assessment. Continuous-wave Doppler allows quantification of LVOT gradients, while colour Doppler evaluates associated mitral regurgitation. The detection of SAM of the mitral valve and its interaction with the hypertrophied septum provides important mechanistic information regarding obstruction [30]. Assessment of diastolic function is also a fundamental component of echocardiographic evaluation in HCM. Parameters including mitral inflow velocities, tissue Doppler-derived early diastolic mitral annular velocity (e′), the E/e′ ratio, LA volume index, and pulmonary artery pressure estimates provide indirect measures of LV filling pressures and disease burden [29]. However, conventional echocardiography has limitations. Image quality is dependent on acoustic windows and operator expertise, and certain phenotypes, including apical HCM, anterolateral hypertrophy, and focal hypertrophy, may be incompletely visualized. In such cases, CMR provides complementary or definitive anatomical assessment [31].

### 3.2. Stress Echocardiography

Dynamic LVOT obstruction is a hallmark of HCM and may be absent at rest despite clinically significant symptoms. Exercise stress echocardiography is therefore an essential component of evaluation in symptomatic patients with no significant resting obstruction. It provides simultaneous assessment of LVOT gradients, exercise-induced mitral regurgitation, blood pressure response, and symptom reproduction [32]. The physiological basis of stress-induced obstruction involves increased contractility, reduced ventricular cavity size, and enhanced Venturi and drag forces contributing to SAM of the mitral valve. A peak LVOT gradient ≥ 50 mmHg during exercise is clinically relevant, particularly when considering septal reduction therapy [3]. Exercise echocardiography has demonstrated clinical value by identifying patients with latent obstruction and explaining symptoms that may otherwise remain unexplained. It also provides prognostic information, as exercise-induced obstruction and abnormal blood pressure response have been associated with increased morbidity in selected patients [33]. Pharmacological stress testing with dobutamine is generally discouraged for routine evaluation of HCM because it may provoke gradients that do not reflect physiological exercise conditions and can produce false-positive results. Exercise-based protocols are preferred when feasible [3].

### 3.3. Three-Dimensional Echocardiography

Three-dimensional (3D) echocardiography provides volumetric assessment of cardiac chambers and improves anatomical visualization compared with conventional two-dimensional imaging. In HCM, 3D echocardiography may enhance evaluation of LV morphology, hypertrophy distribution, mitral valve anatomy, and papillary muscle abnormalities [34].

One important advantage of 3D echocardiography is improved assessment of LV volumes and ejection fraction, reducing geometric assumptions inherent to two-dimensional methods. This may be particularly relevant in HCM, where abnormal ventricular geometry can limit the accuracy of conventional measurements [35]. Three-dimensional imaging also contributes to procedural planning by providing detailed visualization of the mitral valve apparatus and subvalvular structures. This information may help identify patients in whom LVOT obstruction is driven not only by septal hypertrophy but also by elongated mitral leaflets or abnormal papillary muscle insertion [18]. Despite these advantages, widespread implementation remains limited by lower temporal resolution compared with two-dimensional echocardiography, dependence on image quality, and limited availability of expertise.

### 3.4. Speckle-Tracking Echocardiography

Speckle-tracking echocardiography (STE) has become an important tool for detecting subtle myocardial dysfunction in HCM patients with preserved LV ejection fraction (LVEF). Global longitudinal strain (GLS), derived from tracking myocardial deformation during the cardiac cycle, identifies impaired longitudinal shortening caused by hypertrophy, fibrosis, and myocardial disarray [36,37]. Reduced GLS is frequently observed in HCM despite preserved LVEF and has been associated with increased myocardial fibrosis, adverse remodelling, heart failure progression, and ventricular arrhythmias [38]. The regional distribution of strain abnormalities may also provide mechanistic information, with reduced deformation commonly observed in hypertrophied segments. STE may have particular value in early disease detection, including genotype-positive individuals without overt hypertrophy, although its role in routine clinical decision-making remains under investigation. Serial assessment of GLS may also provide a sensitive marker of disease progression and response to therapy [38]. Limitations include variability related to vendor-specific software, loading conditions, image quality, and differences in acquisition protocols. Standardization remains an important challenge before strain parameters can be universally incorporated into clinical risk models.

### 3.5. Left Atrial Strain

LA remodelling is a major consequence of chronic elevation of LV filling pressures in HCM and is strongly associated with atrial fibrillation (AF), stroke, and heart failure symptoms. Conventional assessment relies mainly on LA volume; however, LA strain provides additional information regarding atrial mechanical function and reservoir properties [39]. LA strain analysis evaluates the three phases of atrial function: reservoir, conduit, and contractile phases. Reduced LA reservoir strain reflects impaired atrial compliance and increased ventricular filling pressures and may precede measurable LA enlargement [40]. Several studies have demonstrated associations between reduced LA strain and incident AF, exercise intolerance, diastolic dysfunction severity, and adverse outcomes in HCM [41]. Because LA strain integrates the cumulative effects of LV stiffness and myocardial remodelling, it may represent an early marker of disease progression. Nevertheless, clinical adoption remains limited by differences in methodology, reference values, and software platforms. Further prospective studies are needed to establish its incremental value over conventional LA parameters.

### 3.6. Myocardial Work Analysis

Myocardial work analysis is an emerging echocardiographic technique that combines LV strain measurements with estimated LV pressure curves to provide load-adjusted indices of myocardial performance. Unlike GLS alone, myocardial work incorporates afterload and ventricular pressure generation, potentially providing a more physiologically meaningful assessment of myocardial function in various clinical scenarios [42,43]. In HCM, myocardial work analysis has demonstrated abnormalities in patients with preserved LVEF and may provide additional information regarding mechanical inefficiency, dyssynchrony, and disease severity [44,45]. Alterations in myocardial work indices have been associated with hypertrophy burden, fibrosis markers, and functional limitation. The clinical relevance of myocardial work in HCM remains investigational, and its role in routine risk stratification requires validation in larger longitudinal cohorts.

### 3.7. Strengths and Limitations of Echocardiography in HCM

The major strengths of echocardiography are its accessibility, portability, absence of radiation exposure, excellent temporal resolution, and unique ability to assess dynamic hemodynamics. It remains the most practical modality for initial diagnosis, family screening, serial follow-up, and assessment of LVOT obstruction.

However, echocardiography has limitations, particularly regarding tissue characterization and detection of complex phenotypes. It cannot directly quantify myocardial fibrosis, and image quality may limit diagnostic accuracy in some patients. Furthermore, advanced echocardiographic parameters such as GLS, LA strain, and myocardial work require further standardization before they can be universally integrated into clinical algorithms. Therefore, echocardiography should be considered the foundation of HCM imaging, complemented by CMR and other modalities when additional anatomical, tissue, or prognostic information is required. The optimal approach is not replacement of echocardiography by advanced imaging, but integration of echocardiographic findings within a broader multimodality framework.

## 4. Cardiac Magnetic Resonance

Cardiac magnetic resonance has become an essential component of contemporary hypertrophic cardiomyopathy evaluation because it provides a comprehensive assessment of cardiac morphology, ventricular function, tissue composition, and myocardial mechanics within a single examination. While echocardiography remains the first-line imaging modality due to its accessibility and ability to assess dynamic hemodynamics, CMR offers unique diagnostic and prognostic information through superior spatial resolution and advanced tissue characterization techniques. Current guidelines recommend CMR in patients with suspected or established HCM when echocardiographic findings are inconclusive, when additional phenotypic characterization is required, and for sudden cardiac death (SCD) risk assessment [2,3].

The increasing importance of CMR in HCM reflects a shift from a purely morphological definition of disease toward a more comprehensive understanding of myocardial remodelling. Beyond quantifying hypertrophy, CMR can identify focal and diffuse fibrosis, detect atypical phenotypes, characterize disease progression, and provide imaging biomarkers associated with adverse outcomes. The integration of late gadolinium enhancement, parametric mapping, strain analysis, and emerging flow-based techniques has positioned CMR as a central tool in precision phenotyping and individualized risk assessment.

### 4.1. Morphological Phenotyping

Accurate characterization of LV morphology is fundamental to the diagnosis and classification of HCM. CMR provides highly reproducible measurements of ventricular volumes, myocardial mass, wall thickness, and regional hypertrophy distribution, overcoming some limitations of echocardiography related to acoustic windows and complex ventricular geometry [46]. HCM exhibits substantial morphological heterogeneity, including asymmetric septal hypertrophy, reverse curvature septal morphology, concentric hypertrophy, mid-ventricular obstruction, and isolated apical hypertrophy. CMR is particularly valuable for detecting atypical forms, such as apical HCM, which may be underestimated by transthoracic echocardiography [13]. In addition, CMR enables detailed assessment of structural features associated with disease severity, including apical aneurysm formation, LV cavity obliteration, papillary muscle hypertrophy or displacement, abnormal papillary muscle insertion, myocardial crypts, and right ventricular involvement.

Myocardial crypts, particularly in the basal inferoseptal region, have been described in individuals with sarcomeric mutations and may represent early markers of genotype-positive disease before overt hypertrophy develops [47]. However, their specificity remains limited, and they should be interpreted within the clinical and genetic context. CMR can also provide an accurate assessment of left ventricular (LV) systolic function, which may be particularly useful for patients with a poor acoustic window or suboptimal results from a transthoracic echocardiogram [31].

### 4.2. Late Gadolinium Enhancement

Late gadolinium enhancement is the most established CMR biomarker in HCM and reflects focal replacement fibrosis caused by expansion of the extracellular space following myocardial injury and collagen deposition. In HCM, LGE typically demonstrates a patchy mid-wall distribution within hypertrophied segments, although heterogeneous patterns involving the right ventricular insertion points, septum, and areas of maximal hypertrophy may occur [8].

The presence and extent of LGE provide important prognostic information. Multiple studies have demonstrated an association between increased LGE burden and adverse outcomes, including ventricular arrhythmias, SCD, heart failure progression, and cardiovascular mortality [9,48]. The prognostic impact appears to be related not only to the presence of fibrosis but also to the quantitative extent of scar burden. Chan et al. demonstrated that extensive LGE was associated with increased risk of SCD events in HCM, supporting its incorporation into contemporary risk assessment strategies [9]. Current guidelines recognize extensive LGE (commonly defined as ≥15% of LV mass) as an additional risk marker that may influence decisions regarding primary prevention implantable cardioverter–defibrillator (ICD) implantation, particularly in patients with otherwise-intermediate risk profiles [2,3].

Despite strong prognostic associations, LGE assessment has limitations. Quantification methods vary between centres, thresholding techniques are not fully standardized, and fibrosis burden should not be interpreted in isolation but integrated with clinical risk factors, genetic findings, ventricular function, and other imaging markers.

### 4.3. Native T1 Mapping

Native T1 mapping provides quantitative assessment of myocardial tissue characteristics without the need for contrast administration. In HCM, elevated native T1 values reflect diffuse myocardial abnormalities, including interstitial expansion, fibrosis, inflammation, and potentially changes in myocardial composition [17]. Unlike LGE, which identifies focal replacement fibrosis, native T1 mapping detects diffuse alterations that may occur earlier in the disease process. Studies have demonstrated increased native T1 values in HCM patients, including individuals with preserved ejection fraction and limited or absent LGE [49]. Native T1 mapping may be particularly useful in identifying early myocardial involvement, differentiating HCM from phenocopies, monitoring disease progression, and improving understanding of genotype-positive/phenotype-negative states [17]. Importantly, native T1 values vary according to scanner platform, magnetic field strength, and acquisition sequence, limiting widespread clinical implementation. Standardization of acquisition protocols and establishment of reference ranges remain necessary before routine incorporation into risk models.

### 4.4. Extracellular Volume Quantification

Extracellular volume fraction (ECV) is calculated using pre- and post-contrast T1 measurements combined with haematocrit correction and provides an estimate of diffuse extracellular matrix expansion. In HCM, increased ECV reflects diffuse interstitial fibrosis and adverse myocardial remodelling [50]. Compared with LGE, which identifies focal scar, ECV provides a more quantitative assessment of diffuse fibrosis. Elevated ECV has been associated with increased LV stiffness, impaired diastolic function, reduced myocardial performance, and adverse clinical outcomes [51]. ECV may have particular value in early disease stages, where diffuse fibrosis precedes the development of extensive replacement scar. However, similar to native T1 mapping, limitations include technical variability and incomplete validation of clinically meaningful thresholds.

### 4.5. T2 Mapping

T2 mapping is a quantitative CMR technique that reflects myocardial water content and is primarily used for detection of edema and active myocardial injury. Although inflammation and edema are not dominant features of typical sarcomeric HCM, increased T2 values may identify patients with active myocardial remodelling or superimposed inflammatory processes [52]. Potential applications of T2 mapping in HCM include the identification of active myocardial injury, differentiation of HCM from inflammatory cardiomyopathies, and assessment of disease activity in selected phenotypes [53]. At present, the clinical role of T2 mapping in routine HCM evaluation remains limited, and further studies are required to determine whether it provides incremental prognostic value beyond established markers such as LGE and fibrosis quantification.

### 4.6. Feature-Tracking Strain Analysis

CMR feature-tracking (CMR-FT) enables assessment of myocardial deformation using routinely acquired cine images, without requiring dedicated tagging sequences. Similar to echocardiographic speckle-tracking, CMR strain analysis provides sensitive detection of impaired myocardial mechanics despite preserved LVEF [54]. Reduced global longitudinal strain (GLS) has been demonstrated in HCM patients and correlates with hypertrophy severity, myocardial fibrosis, and functional limitation [38]. Because deformation abnormalities may precede overt systolic dysfunction, CMR-FT may provide early markers of disease progression. CMR-derived strain has several advantages, including excellent reproducibility and independence from acoustic window limitations [54]. However, variability between software platforms and limited standardization currently restrict widespread clinical application.

### 4.7. Four-Dimensional Flow MRI

Four-dimensional (4D) flow MRI is an emerging technique that enables three-dimensional visualization and quantification of intracardiac blood flow throughout the cardiac cycle [15]. In HCM, abnormal flow patterns related to LVOT obstruction, altered ventricular geometry, and mitral valve abnormalities may provide additional mechanistic insights.

Potential applications include the quantification of abnormal flow jets, assessment of LVOT obstruction, evaluation of kinetic energy and viscous energy loss, characterization of vortex formation, and improved understanding of exercise intolerance. Although 4D flow MRI remains primarily a research tool, future applications may include improved phenotyping of obstructive HCM and assessment of response to septal reduction therapies.

## 5. Cardiac Computed Tomography

Cardiac computed tomography (CT) occupies a complementary role within the multimodality imaging evaluation of hypertrophic cardiomyopathy (HCM). Although echocardiography and CMR remain the primary imaging modalities for diagnosis, phenotyping, and risk stratification, cardiac CT provides unique advantages related to its high spatial resolution, rapid acquisition, and excellent visualization of cardiac and extracardiac anatomy [55]. In contemporary practice, CT is primarily used in selected clinical scenarios, including patients with contraindications or limitations to CMR, evaluation of concomitant coronary artery disease, pre-procedural planning for septal reduction therapies, and detailed assessment of complex mitral valve and subvalvular anatomy [7]. Unlike CMR, CT does not provide comprehensive myocardial tissue characterization and involves exposure to ionizing radiation and iodinated contrast. Therefore, it should not be considered a replacement for CMR but rather an imaging complement that addresses specific anatomical and procedural questions. With recent technological developments, including photon-counting CT, the potential applications of CT in HCM are expanding.

### 5.1. Cardiac CT in Patients Unable to Undergo CMR

CMR is considered the reference standard for morphological characterization and myocardial tissue assessment in HCM; however, a proportion of patients cannot undergo CMR because of contraindications, limited availability, severe claustrophobia, inability to cooperate with breath-holding, or the presence of certain implanted devices [56]. In these situations, cardiac CT provides an alternative method for accurate anatomical evaluation. Modern cardiac CT scanners provide excellent spatial resolution and high-quality three-dimensional reconstruction of cardiac structures, allowing assessment of LV hypertrophy distribution, apical morphology, ventricular aneurysm formation, papillary muscle abnormalities, mitral valve anatomy and coronary anatomy. CT may be particularly useful in patients with complex hypertrophic phenotypes where echocardiographic windows are suboptimal and CMR is not feasible. However, the inability of conventional CT to reliably characterize myocardial fibrosis remains a major limitation compared with CMR-based LGE and parametric mapping techniques.

### 5.2. Coronary Artery Evaluation

Although HCM is primarily a myocardial disease, evaluation of the coronary arteries may be clinically relevant because concomitant coronary artery disease (CAD) can contribute to symptoms, myocardial ischemia, and adverse outcomes, particularly in older patients or those with cardiovascular risk factors [57]. Coronary CT angiography (CCTA) provides a non-invasive assessment of coronary anatomy with high diagnostic accuracy for the detection of obstructive coronary disease [58]. In HCM patients presenting with chest pain, exertional symptoms, abnormal stress testing, or unclear ischemic mechanisms, CCTA may help distinguish epicardial coronary disease from HCM-related mechanisms such as microvascular dysfunction. CT may also identify anatomical features relevant to treatment planning, including coronary artery course and the relationship with the hypertrophied myocardium, septal perforator anatomy before alcohol septal ablation, and coronary plaque distribution.

However, it is important to distinguish epicardial coronary disease from the microvascular abnormalities commonly observed in HCM. Coronary microvascular dysfunction, which is associated with myocardial ischemia and adverse remodelling, is better assessed using functional techniques such as positron emission tomography (PET) or stress perfusion CMR [10].

### 5.3. Surgical and Septal Reduction Therapy Planning

In patients with obstructive HCM who remain symptomatic despite medical therapy, septal reduction therapy, including surgical septal myectomy or alcohol septal ablation, may be indicated [3]. Detailed anatomical characterization is essential for selecting the appropriate intervention and optimizing procedural outcomes. Cardiac CT provides high-resolution visualization of the interventricular septum, LVOT anatomy, coronary arteries, and surrounding structures. Three-dimensional CT reconstructions can assist in defining the extent and location of septal hypertrophy, identifying abnormal papillary muscle insertion, assessing accessory papillary muscles, evaluating the relationship between the septum and mitral valve apparatus, and planning alcohol septal ablation by delineating septal perforator anatomy. This information may be particularly valuable in patients with complex obstruction mechanisms in whom LVOT narrowing is not caused solely by basal septal hypertrophy. CT findings can complement echocardiography and CMR by providing a detailed anatomical roadmap for intervention [59].

### 5.4. Assessment of Mitral Valve Anatomy and Subvalvular Abnormalities

Mitral valve abnormalities play an important role in the pathophysiology of obstructive HCM [60]. In addition to septal hypertrophy, obstruction may result from elongated mitral valve leaflets, abnormal papillary muscle position, accessory papillary muscles, and altered mitral-septal geometry. Three-dimensional cardiac CT offers excellent spatial resolution and allows detailed evaluation of mitral leaflet morphology, leaflet length, papillary muscle configuration, subvalvular apparatus, and mitral annular geometry. This information may be particularly useful in patients undergoing surgical myectomy, where concomitant mitral valve intervention may be required. CT can complement echocardiographic assessment by providing a more complete anatomical understanding of the mechanisms contributing to LVOT obstruction.

### 5.5. Emerging Role of Photon-Counting CT

Photon-counting CT (PCCT) represents a major technological advancement in cardiovascular CT imaging. Unlike conventional energy-integrating detectors, photon-counting detectors directly convert X-ray photons into electrical signals, enabling improved spatial resolution, reduced electronic noise, and enhanced spectral imaging capabilities [61]. Potential advantages of PCCT in HCM include improved anatomical characterization. Higher spatial resolution may allow more accurate assessment of myocardial thickness, small anatomical structures, papillary muscle abnormalities, and mitral valve morphology. Enhanced spatial resolution may improve visualization of coronary arteries and small septal perforators, facilitating procedural planning. The clinical role of PCCT in HCM remains investigational, and prospective studies are needed to determine whether it provides incremental diagnostic or prognostic value compared with established imaging modalities.

## 6. Nuclear Imaging

Nuclear imaging techniques provide complementary functional and molecular information that cannot be obtained from conventional structural imaging modalities. While echocardiography and cardiac magnetic resonance (CMR) remain the primary imaging techniques for diagnosis and phenotyping of hypertrophic cardiomyopathy (HCM), nuclear imaging contributes unique insights into myocardial perfusion, coronary microvascular function, metabolism, and tissue-specific processes [62]. The current clinical role of nuclear imaging in HCM is selective rather than routine, but advances in positron emission tomography (PET) technology and the development of novel radiotracers may expand its role in disease characterization and personalized risk assessment. The most established application of nuclear imaging in HCM is the assessment of coronary microvascular dysfunction (CMD) using quantitative myocardial perfusion PET. Additional applications include evaluation of myocardial perfusion abnormalities, differentiation of HCM from phenocopies such as cardiac amyloidosis using bone-avid tracers, and emerging molecular imaging approaches targeting fibrosis, inflammation, and myocardial metabolism.

### 6.1. PET Assessment of Coronary Microvascular Dysfunction

Coronary microvascular dysfunction is an important pathophysiological component of HCM and contributes to myocardial ischemia, fibrosis development, ventricular remodelling, and progression toward heart failure. In HCM, increased myocardial mass, abnormal intramyocardial arteriolar architecture, impaired vasodilatory reserve, and increased extravascular compressive forces may reduce myocardial blood flow despite angiographically normal epicardial coronary arteries [63]. Positron emission tomography (PET) provides a non-invasive technique for quantitative assessment of myocardial blood flow (MBF) and coronary flow reserve (CFR) [64]. The technique allows measurement of absolute resting and stress myocardial perfusion, enabling detection of impaired coronary vasodilatory capacity [65].

Early studies demonstrated that patients with HCM frequently exhibit reduced CFR, particularly in hypertrophied myocardial segments, even in the absence of significant epicardial coronary artery disease. Cecchi et al. demonstrated that impaired coronary microvascular function was associated with increased risk of clinical deterioration and adverse outcomes, establishing CMD as an important contributor to disease progression [66].

Reduced CFR has subsequently been associated with increased myocardial fibrosis, greater hypertrophy burden, ventricular dysfunction, heart failure progression, and adverse cardiovascular outcomes [65,66]. The combination of PET-derived perfusion parameters with CMR markers of fibrosis may provide complementary information regarding the relationship between ischemic injury, myocardial remodelling, and arrhythmogenic risk. Patients with preserved ejection fraction but marked CMD may represent a subgroup at increased risk of progressive disease. Despite these findings, routine PET evaluation of CMD is not currently recommended for all HCM patients, because of limited availability, cost, radiation exposure, and the need for specialized expertise [3]. Its role is primarily reserved for selected patients with unexplained symptoms, suspected ischemic mechanisms, discordant findings between modalities, or research evaluation.

### 6.2. Myocardial Perfusion Imaging

Assessment of myocardial perfusion provides insight into the functional consequences of HCM-related microvascular abnormalities. Conventional single-photon emission computed tomography (SPECT) and PET perfusion imaging have demonstrated that myocardial ischemia may occur in HCM despite normal epicardial coronary arteries. Perfusion abnormalities in HCM are thought to result from inadequate capillary density relative to hypertrophied myocardium, increased myocardial oxygen demand, impaired coronary vasodilatory reserve, and elevated LV end-diastolic pressure causing microvascular compression [63]. Stress perfusion imaging may help explain exertional chest pain and dyspnoea in patients without obstructive coronary artery disease. In addition, abnormalities in perfusion may correlate with areas of myocardial fibrosis identified by CMR, supporting the concept that repeated ischemic injury contributes to adverse remodelling [65]. PET has advantages over SPECT because it provides an absolute quantification of myocardial blood flow, measurement of coronary flow reserve, higher spatial and temporal resolution, and improved diagnostic accuracy for microvascular dysfunction. However, SPECT remains more widely available and may still be used in selected clinical settings, although its role in HCM-specific risk assessment is limited.

### 6.3. Nuclear Imaging in Differential Diagnosis of Hypertrophic Phenotypes

One of the expanding roles of nuclear imaging in patients with LV hypertrophy is differentiation between HCM and infiltrative cardiomyopathies, particularly transthyretin cardiac amyloidosis (ATTR-CM) [67]. Bone-seeking radiotracers have demonstrated high diagnostic accuracy for ATTR-CM when interpreted together with appropriate exclusion of monoclonal protein disorders [68]. This application is particularly relevant because ATTR-CM may mimic HCM by presenting with increased LV wall thickness, diastolic dysfunction, and heart failure symptoms. Multimodality assessment combining echocardiography, CMR tissue characterization, and bone scintigraphy enables accurate differentiation between sarcomeric HCM and infiltrative disease.

## 7. Imaging in Differential Diagnosis of Left Ventricular Hypertrophy

The identification of increased LV wall thickness represents one of the most frequent diagnostic challenges in cardiovascular imaging. Although HCM is the most common inherited cause of pathological LV hypertrophy (LVH), increased wall thickness may also result from physiological adaptation, pressure overload, infiltrative disorders, metabolic diseases, and systemic genetic syndromes. Accurate differentiation between these entities is essential because prognosis, genetic implications, therapeutic strategies, and family screening approaches differ substantially (Table 2).

A multimodality imaging approach combining echocardiography, CMR, CT, and nuclear imaging allow integration of morphological, functional, tissue, and molecular information. Echocardiography provides initial assessment of hypertrophy pattern and hemodynamics, while CMR plays a central role through tissue characterization. CT and nuclear imaging provide complementary information in selected cases, particularly when assessing coronary anatomy, procedural planning, or infiltrative disease.

### 7.1. Athlete’s Heart

Physiological cardiac remodelling associated with intensive athletic training may mimic HCM, particularly in highly trained endurance athletes who develop increased LV wall thickness. Differentiating athlete’s heart from early HCM can be challenging, especially when wall thickness falls within the overlap zone (approximately 13–15 mm) [69]. Echocardiography typically demonstrates symmetrical LV hypertrophy with symmetrically enlarged ventricular cavity size, normal or enhanced diastolic function, normal left atrial size, and absence of significant LVOT obstruction. In contrast, HCM more commonly demonstrates asymmetric hypertrophy, reduced LV cavity size, abnormal diastolic function, and impaired myocardial deformation.

CMR provides additional discriminatory information through assessment of morphology and tissue characteristics. Athlete’s heart is generally associated with absence of significant late gadolinium enhancement, normal native T1 values, normal ECV, and increased LV mass accompanied by increased chamber volume. Conversely, HCM frequently demonstrates focal fibrosis, increased T1 values, and reduced myocardial deformation [70]. Exercise-induced remodelling may regress following detraining, and serial imaging after a period of reduced training intensity may assist diagnosis in selected cases. However, detraining studies are not always practical, and interpretation should incorporate clinical history, family history, genetic findings, and comprehensive imaging assessment.

### 7.2. Hypertensive Heart Disease

Chronic systemic hypertension is one of the most common acquired causes of LVH and may overlap phenotypically with HCM. Hypertensive heart disease (HHD) typically produces concentric LV remodelling due to increased afterload, although asymmetric patterns may occasionally occur. Echocardiographic features favouring HHD include concentric LV hypertrophy, increased relative wall thickness, increased LV mass, and impaired LV relaxation in the absence of marked LVOT obstruction [71]. CMR provides important differentiation through tissue characterization. Compared with HCM, hypertensive LVH usually demonstrates lower fibrosis burden, less frequent LGE, and smaller increases in native T1 and ECV [72]. However, advanced hypertensive disease may produce substantial fibrosis and overlap with HCM phenotypes [73]. Clinical context remains essential. Older age, longstanding hypertension, metabolic risk factors, and regression of hypertrophy after blood pressure control support HHD, whereas family history, marked hypertrophy disproportionate to blood pressure elevation, and sarcomeric variants favour HCM.

### 7.3. Cardiac Amyloidosis

Cardiac amyloidosis represents one of the most important HCM phenocopies because both conditions may present with increased LV wall thickness, diastolic dysfunction, and heart failure symptoms. Differentiation is clinically critical because disease-specific therapies are available for amyloid cardiomyopathy [74]. Echocardiography may demonstrate concentric LV thickening, increased ventricular wall brightness (“sparkling” appearance, although nonspecific), biatrial enlargement, restrictive filling pattern, and reduced global longitudinal strain with relative apical sparing and the “cherry on top” global longitudinal strain pattern [75].

CMR provides characteristic findings of diffuse subendocardial or transmural LGE, abnormal gadolinium kinetics, markedly elevated native T1, and increased ECV. These findings contrast with the patchy mid-wall fibrosis pattern more typical of HCM [76]. Nuclear imaging has transformed the diagnosis of transthyretin cardiac amyloidosis (ATTR-CM). Bone-avid tracers such as 99mTc-PYP, 99mTc-DPD, and 99mTc-HMDP demonstrate myocardial uptake in ATTR-CM and may permit non-invasive diagnosis when plasma cell dyscrasia has been excluded [68]. Multimodality integration is essential because ATTR-CM can coexist with other causes of LVH, particularly in elderly patients.

### 7.4. Fabry Disease

Fabry disease is an X-linked lysosomal storage disorder caused by deficiency of α-galactosidase A, resulting in intracellular accumulation of globotriaosylceramide. Cardiac involvement frequently manifests as LVH and may closely mimic HCM [77]. Echocardiography typically demonstrates concentric LVH with increased papillary muscle size, and LV diastolic dysfunction. A distinctive CMR feature is reduced native T1, reflecting intracellular lipid accumulation, particularly in early disease stages. As disease progresses, focal fibrosis may develop, typically involving the inferolateral basal wall with mid-wall or subepicardial LGE [78]. The combination of unexplained LVH, low native T1 values, inferolateral fibrosis, and systemic features (renal disease, neuropathy, angiokeratomas) should prompt evaluation for Fabry disease. Early diagnosis is important because enzyme replacement therapy and chaperone therapy may alter disease progression.

### 7.5. Glycogen Storage Diseases

Several glycogen storage disorders can produce severe LVH, particularly in children and young adults. These include Pompe disease and PRKAG2 syndrome. Pompe disease results from lysosomal acid α-glucosidase deficiency and causes glycogen accumulation within cardiomyocytes [79]. Imaging findings include marked concentric LVH, increased LV mass, and variable systolic dysfunction. CMR findings are variable and may include increased native T1 and patchy fibrosis in advanced disease [11]. Clinical features such as skeletal muscle weakness, respiratory involvement, and elevated muscle enzymes assist diagnosis.

PRKAG2 syndrome is characterized by abnormal glycogen metabolism due to mutations affecting AMP-activated protein kinase signalling. Characteristic features include LVH resembling HCM, ventricular pre-excitation, conduction abnormalities, and atrioventricular block [80]. CMR generally demonstrates hypertrophy without the extensive fibrosis typical of sarcomeric HCM. Recognition of this phenotype is important because management differs substantially from conventional HCM.

### 7.6. Danon Disease

Danon disease is an X-linked lysosomal storage disorder caused by mutations in the LAMP2 gene [81]. It frequently presents with severe LV hypertrophy that develops rapidly, resulting in wall thicknesses exceeding 30 mm, particularly in young males. Imaging features include severe concentric or asymmetric left ventricular hypertrophy, rapid progression to systolic dysfunction and LV dilation in advanced disease. CMR may demonstrate extensive late gadolinium enhancement, increased myocardial fibrosis, and abnormal myocardial mechanics [82]. Furthermore, clinical features of Danon disease include skeletal myopathy, cognitive impairment and elevated creatine kinase levels, as well as early disease onset. Recognizing Danon disease is essential because the prognosis is often more severe than that of sarcomeric HCM and advanced heart failure therapies may need to be considered earlier [83].

### 7.7. Mitochondrial Cardiomyopathies

Mitochondrial disorders represent a heterogeneous group of diseases affecting oxidative phosphorylation and myocardial energy metabolism [84]. Cardiac involvement may present with hypertrophy, dilation, arrhythmias, or mixed phenotypes. Imaging findings are variable with concentric LVH, impaired systolic function, abnormal strain patterns, and increased myocardial fibrosis in advanced stages. CMR may demonstrate patchy non-ischemic LGE, altered native T1 and ECV, and evidence of metabolic remodelling. The presence of extracardiac manifestations, including neurological abnormalities, ophthalmological disease, diabetes, or skeletal muscle involvement, is often crucial for diagnosis.

### 7.8. Cardiac Sarcoidosis

Cardiac sarcoidosis is an inflammatory cardiomyopathy that may mimic HCM through regional hypertrophy, wall thickening, ventricular dysfunction, and arrhythmias [85]. Recognition is important because immunosuppressive therapy may modify disease progression. Echocardiographic findings may include regional wall motion abnormalities, ventricular aneurysms, reduced LV function and basal septal thinning. CMR is central for diagnosis and typically demonstrates patchy mid-wall or subepicardial LGE, involvement of the basal septum and lateral wall, and myocardial edema on T2 mapping [86]. FDG-PET provides complementary information by identifying active inflammatory myocardial metabolism [87].

The combination of CMR fibrosis assessment and FDG-PET inflammatory assessment allows differentiation between active inflammation requiring treatment and chronic scar substrate associated with arrhythmic risk.

## 8. Imaging-Based Risk Stratification in Hypertrophic Cardiomyopathy

Risk stratification in HCM has evolved from a predominantly clinical approach toward a multimodality imaging-based strategy integrating markers of myocardial remodelling, fibrosis, hemodynamic burden, and electrical instability [88]. Traditional risk models based on clinical characteristics such as family history of sudden cardiac death (SCD), unexplained syncope, maximal wall thickness, LVOT obstruction, age, and ventricular function remain important; however, they do not fully capture the biological heterogeneity of HCM [2,3].

Advanced imaging has substantially improved the ability to identify patients at increased risk of adverse outcomes (Figure 2). Echocardiography provides essential information regarding hypertrophy, obstruction, diastolic dysfunction, and chamber remodelling. CMR contributes unique tissue characterization through LGE, native T1 mapping, ECV, and strain analysis (Table 3). Cardiac CT provides complementary anatomical information, particularly for coronary anatomy and procedural planning, whereas nuclear imaging, especially PET, provides insights into coronary microvascular dysfunction, inflammation, and myocardial metabolism. A clinically useful approach is to organize imaging biomarkers according to the outcome they predict rather than according to imaging modality. This framework better reflects contemporary precision cardiology and highlights the complementary contribution of each imaging technique.

Sudden cardiac death remains one of the most feared complications of HCM and is primarily related to ventricular tachyarrhythmias arising from myocardial disorganization, fibrosis, and electrical instability. Although absolute SCD risk remains low in many patients, accurate identification of individuals who may benefit from implantable cardioverter–defibrillator (ICD) therapy remains challenging. Echocardiography remains fundamental for initial SCD risk assessment. Established echocardiographic markers include maximal LV wall thickness, LVOT obstruction, LV systolic dysfunction, left ventricular apical aneurysm, and abnormal global longitudinal strain. Marked hypertrophy (commonly ≥30 mm) is associated with increased SCD risk and remains an important risk modifier in current guidelines [3]. LVOT obstruction reflects increased myocardial stress and has been associated with adverse outcomes, although obstruction alone is insufficient to define high arrhythmic risk [89]. Reduced GLS may identify early myocardial dysfunction despite preserved ejection fraction. Several studies have demonstrated that impaired longitudinal deformation correlates with fibrosis burden and arrhythmic risk, suggesting potential incremental value beyond conventional echocardiographic parameters [36].

CMR has become one of the most important imaging tools for refinement of SCD risk assessment because of its ability to characterize myocardial fibrosis. LGE represents replacement fibrosis and is one of the strongest imaging biomarkers associated with ventricular arrhythmias and SCD. Extensive LGE (typically ≥15% of LV mass) is associated with increased risk of malignant ventricular arrhythmias and has been incorporated into contemporary guideline-based risk assessment [90]. Chan et al. demonstrated that quantitative LGE burden provided incremental prognostic information beyond conventional risk markers. Subsequent studies confirmed that fibrosis burden correlates with ventricular arrhythmia risk and adverse outcomes [9]. Other CMR features associated with increased SCD risk include LV apical aneurysm, LVEF <50%, extensive hypertrophy, increased native T1 and ECV, and abnormal myocardial strain. Apical aneurysms represent areas of regional myocardial scarring and are associated with ventricular arrhythmias, thromboembolism, and progressive heart failure [25].

## 9. Imaging-Guided Management in Hypertrophic Cardiomyopathy

The management of HCM has evolved from a symptom-driven approach toward a more individualized strategy integrating clinical characteristics, genetics, electrophysiology, and multimodality imaging (Table 4). Imaging is no longer limited to diagnosis and surveillance but has become central to therapeutic decision-making, including SCD prevention, selection of septal reduction strategies, assessment of response to medical therapy, and longitudinal risk reassessment [3]. Contemporary guidelines emphasize shared decision-making and management within experienced HCM centres, particularly when considering invasive therapies, ICD implantation, or complex phenotypes.

### 9.1. Imaging-Guided ICD Implantation

Prevention of SCD is one of the most important management goals in HCM. Implantable cardioverter–defibrillator (ICD) therapy is effective in terminating malignant ventricular arrhythmias; however, because many HCM patients have a low absolute risk of SCD, accurate identification of individuals likely to benefit remains challenging [91]. Traditional clinical risk markers include previous cardiac arrest or sustained ventricular tachycardia, family history of HCM-related SCD, unexplained syncope, massive LV hypertrophy, LV systolic dysfunction, LV apical aneurysm, and significant LVOT obstruction. Imaging has substantially refined risk assessment by identifying the myocardial substrate responsible for arrhythmogenesis [92].

Echocardiography provides several established markers relevant to ICD consideration. Massive hypertrophy (typically ≥30 mm) is associated with increased SCD risk and remains an important risk modifier in contemporary guidelines. LVEF <50% represents an advanced HCM phenotype and is associated with increased risk of ventricular arrhythmias and heart failure events. Echocardiography can detect apical aneurysms, although CMR is more sensitive. These aneurysms represent areas of fibrosis and electrical instability and are associated with ventricular tachycardia and thromboembolic complications.

CMR has become the most important imaging tool for refining ICD decisions because it identifies the arrhythmogenic substrate directly [93]. LGE reflects replacement fibrosis and is strongly associated with ventricular arrhythmias. Extensive LGE, commonly defined as ≥15% of LV mass, is recognized as an important modifier of SCD risk and may influence ICD decisions, particularly in patients with intermediate clinical risk. Additional CMR findings supporting ICD consideration include extensive myocardial fibrosis, LV apical aneurysm, LVEF reduction, progressive adverse remodelling, and increased extracellular volume fraction.

CMR therefore provides a biological assessment of arrhythmic risk rather than simply measuring anatomical severity.

### 9.2. Imaging-Guided Septal Reduction Therapy

Approximately 20–30% of patients with HCM develop significant LVOT obstruction, which contributes to exertional dyspnea, chest pain, syncope, and heart failure symptoms. When symptoms persist despite optimal medical therapy, septal reduction therapy (SRT) may be considered [94]. Available approaches include surgical septal myectomy and alcohol septal ablation (ASA). Both strategies aim to reduce LVOT obstruction but require detailed anatomical assessment to determine the optimal approach.

#### 9.2.1. Alcohol Septal Ablation

Alcohol septal ablation involves selective injection of ethanol into septal perforator arteries supplying the hypertrophied basal septum, producing a controlled infarction and subsequent thinning of the obstructing myocardium [95]. Echocardiography is central before, during, and after ASA. Pre-procedural assessment includes resting LVOT gradient, provoked obstruction, SAM of the mitral valve, mitral regurgitation, and septal morphology. Intraprocedural echocardiography confirms reduction in LVOT gradient and evaluates complications. Cardiac CT has an expanding role in ASA planning because it provides detailed visualization of septal perforator coronary anatomy, the relationship between septal branches and the hypertrophied myocardium, coronary artery disease, and alternative obstruction mechanisms. CT may help identify patients in whom ASA is anatomically feasible and reduce procedural uncertainty. CMR can characterize procedural success by demonstrating infarct location, scar formation, reduction in septal thickness, and ventricular remodelling. The extent and location of ablation-related scar may also provide prognostic information, although routine post-procedure CMR is not universally required.

#### 9.2.2. Surgical Septal Myectomy

Surgical septal myectomy remains the reference standard of septal reduction therapy, particularly for patients with complex LVOT obstruction, severe hypertrophy, or associated mitral/subvalvular abnormalities [96]. The procedure involves removal of the hypertrophied septal myocardium to enlarge the LVOT and reduce SAM-related obstruction. Echocardiography provides assessment of obstruction mechanism, septal thickness, SAM severity, mitral regurgitation, and exercise-induced gradients. Three-dimensional echocardiography may improve visualization of mitral valve and papillary muscle abnormalities. Furthermore, CMR provides detailed anatomical characterization of hypertrophy distribution, papillary muscle abnormalities, anomalous muscle bundles, and myocardial fibrosis. This is particularly important because obstruction is not always caused by septal hypertrophy alone. High-resolution CT may provide additional anatomical information regarding coronary arteries and septal morphology.

### 9.3. Cardiac Myosin Inhibitors and Imaging-Guided Therapy

The introduction of cardiac myosin inhibitors represents a major therapeutic advance in obstructive HCM [97,98]. These agents reduce excessive actin–myosin cross-bridge formation, decreasing hypercontractility and LVOT obstruction. Mavacamten is currently the best-established agent in this class and has demonstrated improvements in symptoms, LVOT gradients, and functional capacity in patients with obstructive HCM. Imaging plays a central role in patient selection and monitoring [99]. Before initiating therapy, echocardiography evaluates maximal law thickness, LVOT gradient, LVEF, mitral valve function, and ventricular geometry. Because excessive reduction in contractility may cause systolic dysfunction, baseline and serial assessment of LVEF are mandatory. Serial echocardiography evaluates the reduction in LVOT gradient, improvement in symptoms, changes in mitral regurgitation, and maintenance of systolic function. CMR may provide additional information regarding remodelling, although its routine role during myosin inhibitor therapy remains under investigation. Exercise echocardiography may be repeated when symptoms change or when latent obstruction is suspected.

## 10. Emerging Technologies

Artificial intelligence (AI), machine learning (ML), and radiomics are emerging as potentially transformative tools in the imaging-based assessment of HCM. Their principal value lies not in replacing established imaging modalities, but in extracting reproducible quantitative information from large and complex datasets and integrating imaging findings with clinical, electrocardiographic, laboratory, and genetic information [100]. In HCM, where phenotypic expression is highly heterogeneous and clinically relevant information is distributed across multiple imaging parameters, AI-based approaches may facilitate automated phenotyping, improve diagnostic consistency, and support individualized risk assessment. Nevertheless, most currently available AI applications remain investigational, and prospective validation demonstrating incremental clinical utility is required before widespread implementation.

One of the most utilized applications of AI is the automated detection and quantification of LV hypertrophy. Deep learning algorithms applied to echocardiographic and CMR images can potentially identify abnormal myocardial thickness, characterize the distribution and geometry of hypertrophy, and distinguish focal from diffuse or atypical patterns [101]. Automated wall-thickness measurements may reduce interobserver variability and facilitate detection of subtle or regional hypertrophy, particularly in anatomically challenging regions such as the LV apex. Such approaches may be particularly useful in large-scale screening or longitudinal assessment, although their performance depends strongly on image quality, acquisition protocols, segmentation accuracy, and the populations used for algorithm development [102].

Automated chamber segmentation and volumetric analysis represent another important application. AI-based segmentation can rapidly delineate the LV, right ventricle, atria, myocardium, and blood pool on echocardiographic and CMR datasets, enabling automated calculation of ventricular volumes, ejection fraction, LV mass, atrial volume, and other geometric parameters. These capabilities could facilitate high-throughput analysis and longitudinal monitoring, although automated outputs still require appropriate quality control and, in many clinical settings, expert verification.

Radiomics provides a complementary approach by converting medical images into large numbers of quantitative features describing intensity, texture, spatial distribution, shape, and tissue heterogeneity [103]. In HCM, radiomic analysis of CMR may identify subtle patterns of myocardial heterogeneity that are not apparent on visual assessment and may complement conventional LGE quantification and parametric mapping. Radiomic features derived from cine, LGE, T1-, T2-, or extracellular-volume maps could potentially characterize different patterns of myocardial remodelling and fibrosis and identify imaging phenotypes associated with adverse outcomes. However, radiomic signatures are highly dependent on image acquisition, reconstruction, segmentation, preprocessing, and feature-extraction methods. Consequently, apparently promising signatures require rigorous standardization and independent validation before they can be considered clinically robust biomarkers.

Another important area is machine learning-based prediction of clinical outcomes [104]. Conventional HCM risk assessment relies on a combination of clinical, family-history, electrocardiographic, genetic, and imaging variables. ML algorithms may identify nonlinear relationships and interactions among these variables that are difficult to capture using conventional statistical models. Imaging-derived features could therefore be incorporated into models predicting SCD, ventricular arrhythmias, and HF progression. Such models may eventually provide individualized estimates of risk rather than relying solely on categorical risk markers. AI may also contribute to the differentiation of HCM from phenocopies. AI algorithms integrating morphology, tissue characterization, strain, perfusion, and clinical or laboratory variables may identify combinations of features characteristic of specific disease entities. In particular, multimodal models could potentially recognize phenotypes that are difficult to distinguish and direct patients toward targeted biochemical, genetic, or nuclear imaging investigations.

Overall, AI, ML, and radiomics have the potential to transform HCM imaging from predominantly qualitative or manually derived assessment toward automated, quantitative, multimodal phenotyping and individualized risk prediction. However, despite these opportunities, several important limitations currently restrict the clinical translation of AI in HCM. These technologies should currently be considered complementary and predominantly investigational. Their widespread clinical implementation should require robust prospective external validation, reproducibility across institutions and imaging platforms, appropriate explainability, a demonstration of incremental value over established clinical and imaging approaches, and—most importantly—evidence of clinical utility and improved patient care.

## 11. Conclusions

Multimodality imaging has transformed the evaluation and management of hypertrophic cardiomyopathy, shifting the clinical approach from a purely morphological diagnosis toward comprehensive phenotypic, functional, and biological characterization. Echocardiography remains the cornerstone of initial diagnosis, hemodynamic assessment, and longitudinal surveillance because of its accessibility and unique ability to evaluate dynamic left ventricular outflow tract obstruction. However, the complex and heterogeneous nature of HCM requires complementary information that can only be obtained through advanced imaging techniques.

The future of HCM imaging will depend on integration rather than replacement of individual modalities. Combining echocardiographic hemodynamics, CMR tissue biomarkers, CT anatomical modelling, and PET molecular information has the potential to create individualized imaging profiles that more accurately predict sudden cardiac death, heart failure progression, arrhythmias, and thromboembolic complications. Emerging technologies, including artificial intelligence, radiomics, machine learning, and multiparametric imaging approaches, may further enhance diagnostic precision and enable earlier detection of disease before overt clinical manifestations.

## Figures and Tables

**Figure 1:**
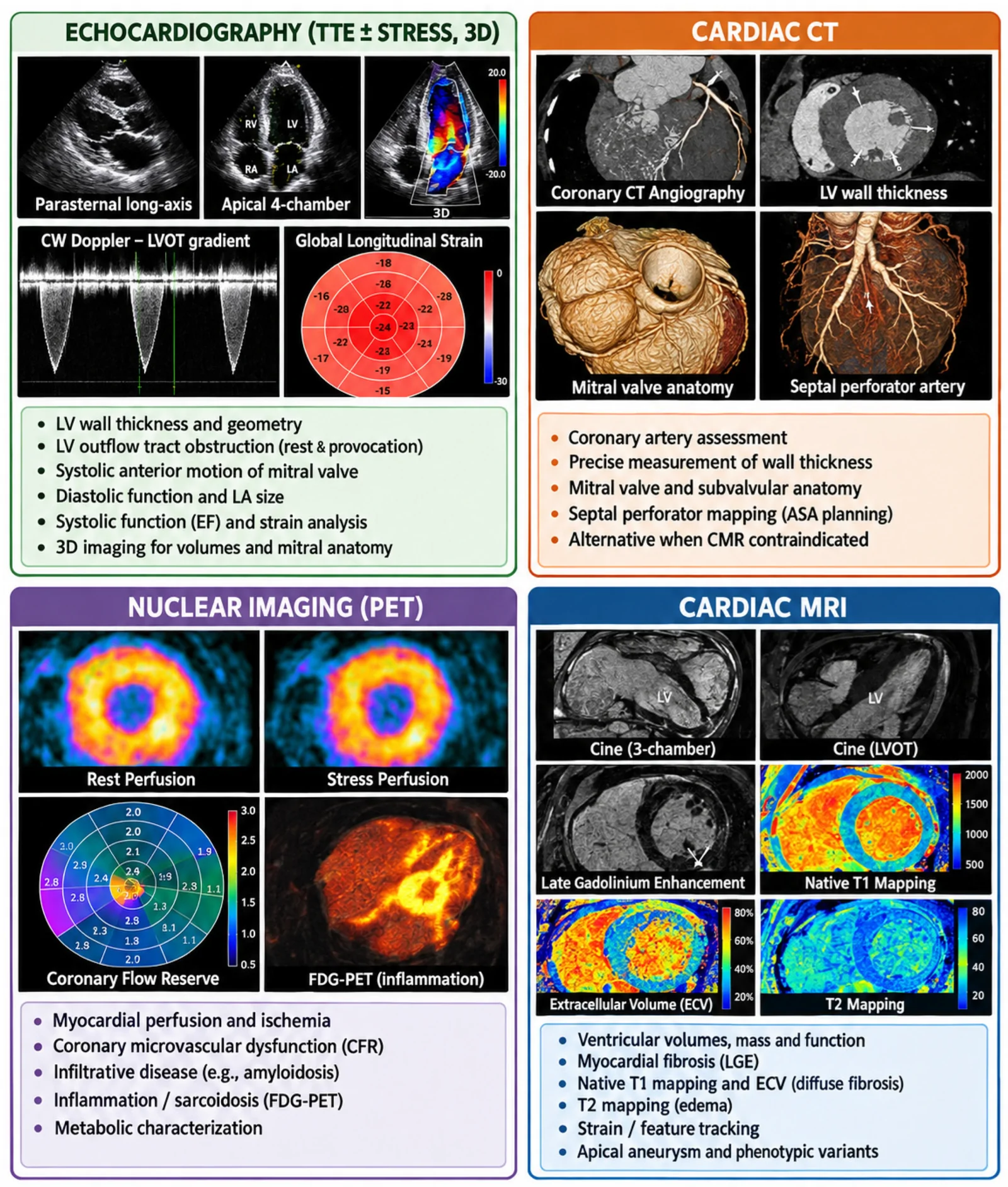
Non-invasive imaging modalities for the evaluation of patients with hypertrophic cardiomyopathy and main findings. TTE—transthoracic echocardiography; 3D—three dimensional; LV—left ventricular; LA—left atrium; EF—ejection fraction; CT—computed tomography; ASA—alcohol septal ablation; CMR—cardiac magnetic resonance; PET—positron emission tomography; CFR—coronary flow reserve; MRI—magnetic resonance imaging; LVOT—left-ventricular outflow tract; LGE—late gadolinium enhancement; ECV—extracellular volume.

**Figure 2:**
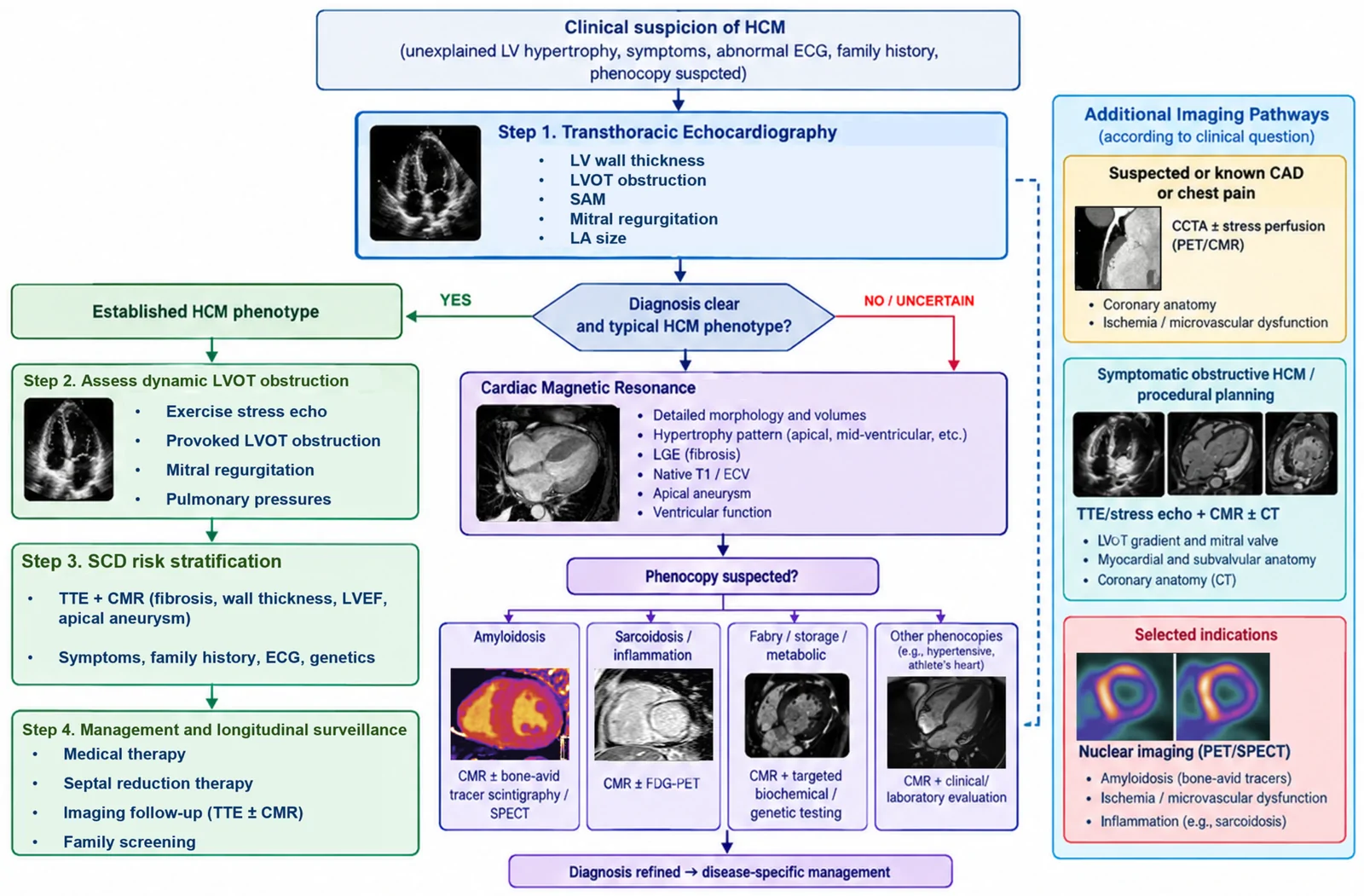
Multimodality imaging algorithm for patients with suspected or confirmed hypertrophic cardiomyopathy. HCM—hypertrophic cardiomyopathy; LV—left ventricular; ECG—electrocardiogram; LVOT—left ventricular outflow tract; SAM—systolic anterior motion; RV—right ventricle; LGE—late gadolinium enhancement; TTE—transthoracic echocardiography; CCTA—cardiac computed tomography; PET—positron emission tomography.

**Table 1:** Strengths and limitations of multimodality imaging techniques in hypertrophic cardiomyopathy.

Imaging Modality	Principal Diagnostic Applications	Major Strengths	Limitations
TTE	First-line diagnosis; assessment of LV wall thickness and morphology; dynamic assessment of LVOT obstruction; systolic and diastolic function; mitral valve abnormalities; LA size.	Widely available, inexpensive, portable, real-time imaging; excellent temporal resolution; dynamic assessment during physiological maneuvers and exercise; no ionizing radiation.	Image quality dependent on acoustic window and operator expertise; limited visualization of apical, anterolateral, and right ventricular hypertrophy; limited tissue characterization.
Advanced echo (3D, speckle-tracking, myocardial work)	Quantitative assessment of ventricular mechanics and atrial function; early detection of myocardial dysfunction.	Detects subclinical systolic dysfunction despite preserved ejection fraction; provides global longitudinal strain, mechanical dispersion, left atrial strain, and myocardial work indices; useful for serial follow-up.	Limited standardization across vendors; dependent on image quality; prognostic thresholds remain under investigation; requires dedicated software and expertise.
CMR	Comprehensive assessment of morphology, ventricular function, hypertrophy distribution, apical aneurysm, papillary muscles, myocardial fibrosis, edema, and tissue characterization.	Gold standard for ventricular volumes and mass; superior spatial resolution; excellent tissue characterization using LGE, native T1, ECV, and T2 mapping; identifies phenotypes missed by echocardiography.	Limited availability; higher cost; contraindications in patients with non-MR-compatible devices or severe claustrophobia; gadolinium use may be restricted in advanced renal impairment; longer examination time.
CCT	Alternative anatomical imaging when CMR is contraindicated; coronary artery evaluation; preprocedural planning for septal reduction therapies; mitral valve and papillary muscle assessment.	High spatial resolution; rapid acquisition; excellent visualization of coronary arteries, cardiac anatomy, and extracardiac structures; useful for procedural planning and three-dimensional reconstruction	Exposure to ionizing radiation and iodinated contrast; limited tissue characterization compared with CMR; lower temporal resolution than echocardiography.
Nuclear imaging (PET/SPECT)	Assessment of myocardial perfusion, coronary microvascular dysfunction, myocardial metabolism, and selected differential diagnoses (e.g., infiltrative cardiomyopathies)	Quantitative measurement of myocardial blood flow and coronary flow reserve; sensitive detection of microvascular dysfunction; functional and metabolic assessment beyond structural imaging	Limited availability; ionizing radiation; relatively low spatial resolution; high cost; not recommended for routine evaluation of HCM

TTE, transthoracic echocardiography; CMR, cardiac magnetic resonance; CCT, cardiac computed tomography; ECV, extracellular volume fraction; LGE, late gadolinium enhancement; LV, left ventricular; LVOT, left ventricular outflow tract; PET, positron emission tomography; SPECT, single-photon emission computed tomography.

**Table 2:** Differential diagnosis of left ventricular hypertrophy using multimodality imaging.

Condition	Echocardiography	CMR	Cardiac CT	Nuclear Imaging	Key Distinguishing Features
Hypertrophic cardiomyopathy	Asymmetric septal hypertrophy, dynamic LVOT obstruction, SAM of the mitral valve, diastolic dysfunction	Patchy mid-wall LGE, elevated native T1 and ECV, apical aneurysm, crypts, papillary muscle abnormalities	Useful when CMR is contraindicated; delineates coronary anatomy and anatomy for septal reduction planning	PET may demonstrate coronary microvascular dysfunction	Familial disease with heterogeneous patterns of hypertrophy and myocardial fibrosis
Hypertensive heart disease	Concentric LVH, mild LA enlargement, absence of SAM or significant LVOT obstruction	Mild diffuse fibrosis, limited or absent LGE, lower native T1/ECV than HCM, regression with blood pressure control	Coronary artery assessment when clinically indicated	Limited role	History of hypertension and concentric remodelling
Athlete’s heart	Symmetric LVH with enlarged LV cavity, normal or supranormal diastolic function, regression after detraining	No LGE (or minimal insertion-point LGE), normal native T1 and ECV	Rarely indicated	Normal myocardial perfusion	Physiological adaptation with preserved myocardial tissue characteristics
Cardiac amyloidosis	Concentric LVH, biatrial enlargement, thickened valves, restrictive filling, reduced GLS with apical sparing	Diffuse subendocardial or transmural LGE, markedly elevated native T1 and ECV	May demonstrate myocardial thickening and extracardiac calcification	Bone scintigraphy identifies transthyretin amyloidosis; PET has a limited role	Multisystem disease with characteristic tissue characterization and radionuclide uptake
Fabry disease	Concentric or asymmetric LVH, prominent papillary muscles, possible valve thickening	Low native T1, inferolateral mid-wall LGE in advanced disease, modest ECV increase	Limited role	PET mainly investigational	Low native T1
Cardiac sarcoidosis	Regional wall motion abnormalities, basal septal thinning, aneurysms	Patchy multifocal LGE (subepicardial or mid-myocardial), myocardial edema on T2	Limited role	FDG-PET demonstrates active myocardial inflammation	Ventricular arrhythmias or conduction disease often accompanies imaging abnormalities
Danon disease	Marked concentric or asymmetric LVH, early systolic dysfunction	Extensive LGE, elevated native T1, diffuse fibrosis	Limited role	Limited role	Present in young patients with skeletal myopathy and cognitive impairment
PRKAG2 syndrome	Concentric LVH with ventricular pre-excitation in many patients	Minimal fibrosis despite marked hypertrophy	Limited role	Limited role	Associated with Wolff–Parkinson–White syndrome and conduction abnormalities
Pompe disease (glycogen storage disease type II)	Concentric LVH, ventricular dysfunction	Variable LGE, elevated T1 in advanced disease	Limited role	Limited role	Skeletal muscle weakness and enzyme deficiency aid diagnosis
Mitochondrial cardiomyopathy	Variable hypertrophy	Patchy non-ischemic LGE, variable T1 and ECV abnormalities	Limited role	May demonstrate altered myocardial metabolism	Multisystem disorder with neurological, ophthalmological, or metabolic manifestations
Aortic stenosis	Concentric LVH, calcified aortic valve, elevated transvalvular gradients	Diffuse fibrosis and variable LGE in advanced disease	Excellent assessment of valve calcification and anatomy	Limited role	Severe valvular obstruction explains hypertrophy
Infiltrative/storage diseases (other)	Variable concentric hypertrophy and diastolic dysfunction	Disease-specific tissue characterization depending on etiology	May identify extracardiac manifestations	Tracer-dependent	Clinical context and multimodality imaging guide diagnosis

CMR, cardiac magnetic resonance; CT, computed tomography; ECV, extracellular volume fraction; FDG, fluorodeoxyglucose; LGE, late gadolinium enhancement; LVH, left ventricular hypertrophy; LVOT, left ventricular outflow tract; PET, positron emission tomography; SAM, systolic anterior motion.

**Table 3:** Imaging biomarkers and their association with clinical outcomes in hypertrophic cardiomyopathy.

Imaging Biomarker	Imaging Modality	Clinical Significance	Associated Adverse Outcomes	Evidence/Comments
Maximum LV wall thickness	Echocardiography, CMR	Marker of disease severity	SCD, ventricular arrhythmias, heart failure progression	Wall thickness ≥ 30 mm is a major SCD risk marker incorporated into current guidelines [3].
LVOT gradient	Echocardiography (rest and stress)	Reflects dynamic obstruction and hemodynamic burden	Heart failure symptoms, reduced exercise capacity, progression to septal reduction therapy	Exercise echocardiography is recommended to identify latent obstruction [7].
LV ejection fraction	Echocardiography, CMR	Marker of systolic function	Advanced heart failure, SCD, cardiovascular mortality	Reduced LVEF (<50%) is associated with poor prognosis [3].
Global longitudinal strain	Speckle-tracking echocardiography, CMR feature tracking	Detects subclinical systolic dysfunction despite preserved LVEF	Ventricular arrhythmias, heart failure progression, adverse remodelling	Reduced GLS has incremental prognostic value beyond LVEF [7].
Left atrial volume	Echocardiography, CMR	Marker of chronic diastolic dysfunction	AF, stroke	Reflects long-term elevation of LV filling pressures [41].
Left atrial strain	Speckle-tracking echocardiography	Sensitive marker of atrial dysfunction	AF, thromboembolism, HF	Emerging biomarker with increasing evidence [41].

CMR, cardiac magnetic resonance; GLS, global longitudinal strain; LV, left ventricular; LVEF, left ventricular ejection fraction; LVOT, left ventricular outflow tract; SCD, sudden cardiac death.

**Table 4:** Clinical scenarios and suggested imaging modalities.

Clinical Scenario	Fist-Line Imaging	Additional Imaging
Scenario 1 Suspected HCM with equivocal echocardiography	TTE identifies increased LV wall thickness but provides limited visualization of the apex.	CMR confirms the distribution and extent of hypertrophy and identifies associated tissue
Scenario 2 Symptomatic patient with suspected dynamic LVOT obstruction	Resting TTE demonstrates limited obstruction	Exercise stress echocardiography reveals a marked increase in LVOT gradient and associated mitral regurgitation, directly influencing therapeutic decision-making.
Scenario 3 HCM with elevated SCD risk	TTE demonstrates hypertrophy and preserved systolic function	CMR additionally demonstrates extensive LGE and/or an apical aneurysm, providing important additional risk information for integrated SCD assessment.
Scenario 4 HCM versus phenocopy	TTE demonstrates increased LV wall thickness	CMR tissue characterization suggests an infiltrative or storage disorder, prompting targeted laboratory/genetic evaluation and, in selected cases, nuclear imaging.
Scenario 5 Pre-procedural assessment	TTE defines LVOT hemodynamics	CMR characterizes hypertrophy and mitral/subvalvular anatomy, and CT provides high-resolution anatomical information when required for coronary assessment or procedural planning.

HCM, hypertrophic cardiomyopathy; TTE, transthoracic echocardiography; LV, left ventricular; CMR, cardiac magnetic resonance; LVOT, left ventricular outflow tract; SCD, sudden cardiac death; CT, computed tomography.

## Data Availability

No new data were created or analyzed in this study.
